# Evaluation of a Faraday cup‐style detector as a beam diagnostic system for ultra‐high dose rate (FLASH) electron beams

**DOI:** 10.1002/acm2.70819

**Published:** 2026-09-24

**Authors:** Alan Lopez, Alexander Baikalov, Kevin Liu, Nolan Esplen, Stefan Bartzsch, Emil Schüler

**Affiliations:** ^1^ Department of Radiation Physics The University of Texas MD Anderson Cancer Center Houston Texas USA; ^2^ The University of Texas MD Anderson Cancer Center UTHealth Graduate School of Biomedical Sciences Houston Texas USA; ^3^ Department of Radiation Oncology School of Medicine and Klinikum rechts der Isar Technical University of Munich Munich Germany; ^4^ Institute of Radiation Medicine Helmholtz Zentrum München GmbH German Research Center for Environmental Health Neuherberg Germany

**Keywords:** beam diagnostics, Faraday cup, FLASH radiotherapy, ultra‐high dose rate

## Abstract

**Background:**

Beam diagnostic systems are essential for the development and quality assurance of ultra‐high dose rate (UHDR) radiation sources to enable reliable delivery of FLASH radiotherapy (RT). Critically, suitable beam diagnostic systems for electron FLASH sources are lacking.

**Purpose:**

In this study, we evaluated a Faraday cup–style beam collector (BC), the BC‐145‐Al, on an UHDR electron linac for FLASH‐RT applications.

**Methods:**

The BC performance was benchmarked against a reference alternating‐current current transformer (ACCT) across a wide range of beam currents and pulse structures to test the BC's charge linearity and signal resolution capabilities. Two termination impedances were tested: 200 kΩ and 13 Ω.

**Results:**

The highly time‐resolved signal of the BC was charge‐proportional when terminated at 200 kΩ and current‐proportional when terminated at 13 Ω, agreeing well with the time‐resolved ACCT signal. The cumulative signals from the BC and ACCT showed excellent agreement, maintaining linearity with a deviation of less than ± 0.5% over 50 consecutive pulses (4‐µs pulse width, 120‐Hz pulse repetition frequency [PRF]). The BC/ACCT signal ratio remained stable within ± 0.5% as the PRF increased from 5 to 120 Hz at constant pulse width. Pulse width measurements from both detectors were also consistent within ± 0.5% across all nominal pulse width values (0.5–4 µs). When the charge per pulse was modulated by either increasing the pulse width or decreasing the source‐to‐surface distance (from 32.3 to 19.3 cm), the BC signal remained approximately linear with respect to the ACCT signal; however, the BC/ACCT ratio deviated by up to ± 6% across this range.

**Conclusions:**

The BC produced highly time‐resolved absolute charge measurements of a pulsed UHDR electron beam across a large range of beam parameters. These results support its potential as a beam diagnostic system for both preclinical studies and clinical applications of electron FLASH radiotherapy.

## 1 INTRODUCTION

Ultra‐high dose rate (UHDR) or FLASH radiation therapy (RT) has shown reductions in normal tissue toxicity while maintaining similar tumor control relative to conventional radiation therapy (CONV), a phenomenon referred to as the FLASH effect.^1^, ^2^ FLASH‐RT is characterized by the use of UHDR irradiation, typically defined as mean dose rates exceeding 40 Gy/s.^3^ In contrast, CONV‐RT involves mean dose rates generally ranging from 0.01 to 0.1 Gy/s. However, defining the mean dose rate (> 40 Gy/s) as the sole parameter required to achieve the FLASH effect may be insufficient. The FLASH effect likely depends also on other physical beam parameters such as dose per pulse (DPP), instantaneous dose rate (IDR), pulse repetition frequency (PRF), total irradiation time and total dose. Understanding the influence of these and other beam parameters on the FLASH effect requires their accurate measurement.

The detectors commonly used in CONV‐RT are limited in providing accurate dosimetry or effectively monitoring the UHDR beams of FLASH‐RT.^4^ For instance, ionization chambers, which serve as a reference standard for dosimetry in CONV‐RT,^5^, ^6^ experience saturation effects at the extremely high DPP levels required for FLASH‐RT. Although several studies have demonstrated that charge‐reading corrections are possible,^7^, ^8^, ^9^, ^10^, ^11^ these approaches remain limited by the criteria prescribed by standards.^5^, ^6^ At the same time, efforts are underway to develop prototype ionization chambers to better meet the requirements of FLASH‐RT.^9^ In CONV‐RT, transmission ionization chambers in the head of clinical linear accelerators (linacs) are used to monitor and control real‐time beam parameters such as dose rate, beam flatness, and beam symmetry.^12^ However, in UHDR beamlines, these transmission chambers fail to accurately monitor the beam because of the same saturation effects observed in point‐dose ionization chambers.^13^ Efforts have been made to apply correction factors and to reconfigure these transmission chambers, demonstrating that it is feasible to measure DPPs up to approximately 1 Gy.^14^, ^15^

The current lack of accurate detectors presents a significant limitation for beam monitoring, diagnostics, and dosimetry in both clinical and preclinical FLASH‐RT trials. Solutions that have been proposed or implemented include passive dosimeters such as radiochromic film, which has been widely used to measure dose and spatial dose distribution of UHDR beams because of its dose linearity and lack of dependence on mean dose rate and DPP.^16^, ^17^ Other suitable, passive point‐detectors include alanine and thermoluminescent dosimeters. Some active dosimeters such as scintillators, diamond detectors, and ion chambers have also been modified to exhibit dose linearity and dose‐rate independence at UHDRs.^18^, ^19^, ^20^, ^21^, ^22^ Although active dosimeters such as these are useful for UHDR dosimetry in clinical or preclinical settings, their applicability for UHDR beam diagnostics is often limited due to insufficient time resolution, lack of absolute charge measurements, and saturation effects at the extreme ranges of UHDR beam currents.^13^, ^23^

One validated solution for beam monitoring systems in UHDR charged particle beamlines is the implementation of alternating‐current current transformers (ACCTs), non‐destructive detectors which are capable of resolving the pulse shape of each pulse of the beam, demonstrate linearity with dose, and show no dependence on dose rate or PRF.^24^, ^25^ When placed in the head of UHDR electron beamlines, ACCTs can provide real‐time information on beam output, and dual‐ACCT configurations can provide information on beam energy.^25^ Although ACCTs can be used for beam diagnostic measurements, they are limited to short distances from the source of the beam because of their small aperture widths.

Similarly, optimized Faraday cups (FCs) have been shown to be dose‐rate independent and linear with dose when used in UHDR proton beamlines (synchrocyclotron) at mean dose rates of up to 1000 Gy/s, allowing the beam pulse width (PW) and overall delivery time to be tracked in relation to the charge collected in the FCs.^26^ FCs consist of a conductive collector that intercepts charged particles, typically utilizing a bias voltage to suppress secondary electron escape. Operating the device within a vacuum environment prevents beam‐induced air ionization, ensuring that the collected charge remains directly proportional to the incident beam intensity.^27^ FCs have been used for calibration, commissioning, and quality assurance in CONV‐RT while also showing properties useful for UHDR proton beamlines.^26^, ^28^ Moreover, in UHDR irradiation using very high‐energy electron beams, FCs have been employed for absolute charge measurements in air and resolving the temporal structure of the electron beam.^26^, ^29^ Similarly, FCs have been used to measure the charge per pulse to characterize UHDR electron systems, as well as for in‐beamline energy and beam‐monitoring measurements.^30^, ^31^, ^32^ Faraday cups have also been used in various electron beam applications, such as measuring electron beams from traveling wave tube guns,^33^ metallic material processing,^34^ and optimization of electron beam welding,^35^ among others. However, using FCs in UHDR electron beams can be further explored for applications in FLASH‐RT.

Here, we present a comprehensive characterization of a modified Faraday cup–style beam collector as a beam diagnostic system for FLASH‐RT UHDR electron beams, which, to our knowledge, has not been previously reported. The detector response was characterized in terms of temporal resolution of the radiation pulse, number of pulses (charge accumulation), charge per pulse, pulse width (PW), and pulse repetition frequency. Performance of the BC was compared with that of an ACCT, which served as a reference detector and has been previously characterized for FLASH‐RT UHDR electron beams.

## 2 METHODS

### 2.1 Experimental setup

**FIGURE 1:**
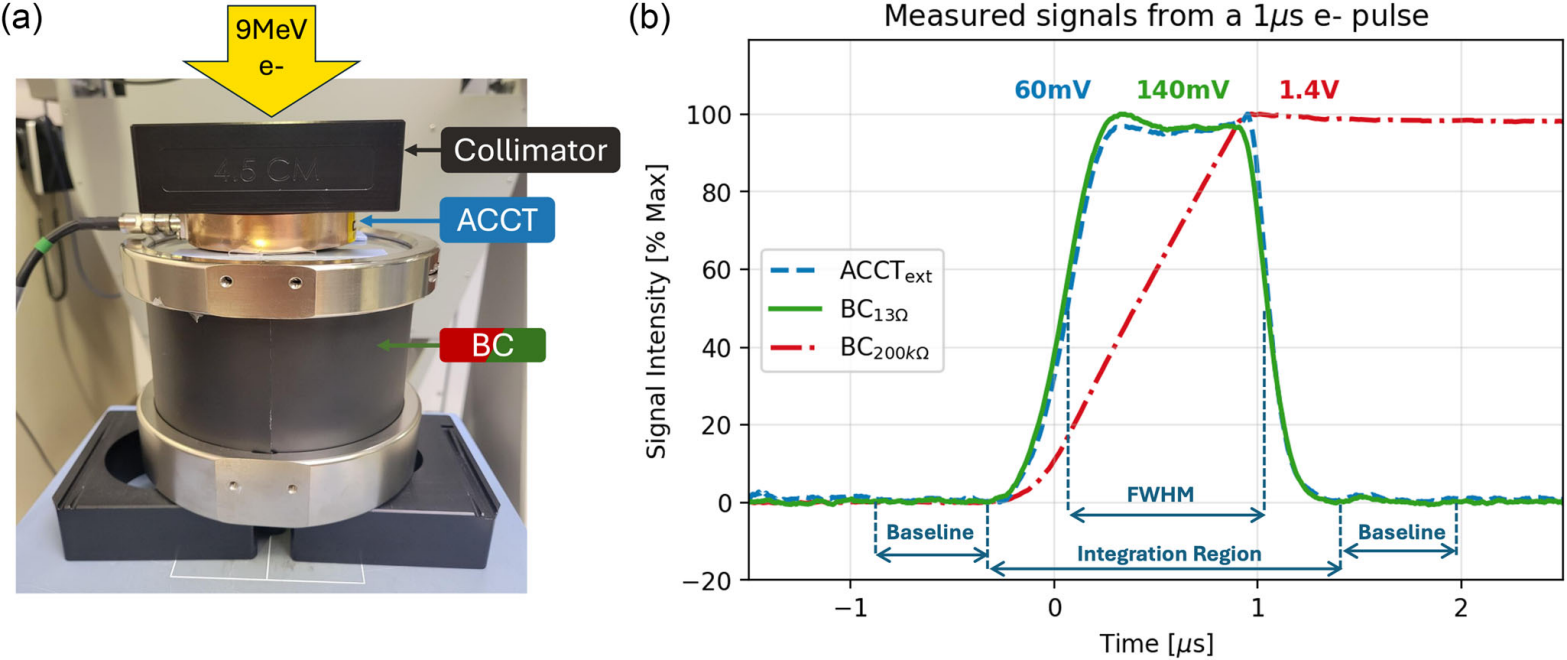
(a) Experimental setup of the beam collector (BC) and alternating‐current current transformer (ACCT) under the linac treatment head. The beam arrives from the top, passing through the 4.5‐cm collimator, through the 5.5‐cm ACCT, and finally impinging on the front surface of the BC. The ACCT is separated from the surface of the BC by two 2‐mm‐thick solid water blocks below the ACCT. The BC is placed on two large plastic supports (black) to allow space for the signal output cable (not visible), located on the bottom surface of the BC. (b) Measured signals from a 1‐µs e‐pulse from the ACCT (dashed line) and the BC terminated at either 13 Ω (solid line) or 200 kΩ (dash‐dot line). The maximum voltage from each signal is indicated on the plot. The signals were first passed through a digital 3‐MHz lowpass filter. The full width at half‐maximum (FWHM), integration region, and baseline regions used to analyze the pulse are indicated below the signal.

The BC‐145‐Al (Pyramid Technical Consultants, Waltham, MA, USA) is a beam collector (BC) designed as a modified Faraday cup. It features a solid aluminum core with layers of dielectric materials specifically designed to capture the primary electrons of the beam while preventing secondary electrons from escaping. Notably, the BC does not require a vacuum system or bias voltage to function. It is cylindrical, with dimensions of 14.25 cm in length and 14.5 cm in diameter. Its solid core is also cylindrical, measuring 10 cm in length and 14.5 cm in diameter. The beam‐incident surface is flat and features a raised lip around the edge. On the opposite surface, a centrally located BNC co‐axial connector enables the collected charge to flow to ground, allowing signal readout. During experiments, the BC signal was terminated with either high impedance (200 kΩ) or low impedance (13 Ω) to find the signal resolution capabilities of the detector. The BC was positioned beneath the treatment head of a 9‐MeV electron UHDR linac (Mobetron, IntraOp, Sunnyvale, CA, USA),^36^ with its incident surface oriented normal to and centered along the beam path (Figure 1a). It was placed on top of two plastic blocks (Figure 1a, black) separated by a gap, allowing the BNC cable to bend and exit laterally from the bottom surface of the BC.

The BC was evaluated against an external ACCT (ACCT‐S‐055‐H, Bergoz Instrumentation, Saint‐Genis‐Pouilly, France) with an inner diameter of 5.5 cm. The generated signal was first processed by an operational amplifier with a dedicated power supply before being read out via a BNC connector. The ACCT signal was terminated with a 50 Ω resistor, as specified by the manufacturer, resulting in a gain factor of 300 mA/V and a rise time of 350 ns. The ACCT was positioned directly atop the BC, resting on two 2‐mm‐thick plastic supports (Figure 1a, light blue) to raise its output cable above the BC's raised lip without interfering with the beam path. This setup created a 2‐mm air gap between the bottom surface of the ACCT and the incident surface of the BC. A 4.5‐cm diameter circular collimator was centered on top of the ACCT to ensure that the collimated beam passed fully through the ACCT and into the incident surface of the BC. At extended source‐to‐surface distance (SSD)—defined here as the distance from the scattering foil to the incident surface of the beam c (BC)—additional collimation was added above the 4.5‐cm collimator to ensure that the full extent of the incident beam passed through the ACCT and into the BC (Figure S1). The ACCT remained fixed relative to the BC throughout all experiments.

GafChromic EBT3 film (Ashland Specialty Ingredients, Bridgewater, NJ, USA) was used to measure dose for some of the experimental conditions. A square piece of film was placed on the surface of the BC, directly centered beneath the ACCT. The film was analyzed 24 hours after irradiation according to a protocol described elsewhere.^37^ The BC signal and film measurements were used to derive the reported dose per pulse and instantaneous dose rate metrics.

### 2.2 Signal analysis

Signals from the BC and ACCT were acquired during beam delivery and digitized with a digital oscilloscope (PicoScope 5000 Series, Pico Technology, St Neots, Cambridgeshire, UK) operating with 14‐bit resolution and sampling rate of 125 MHz. The raw, time‐resolved signal from the BC was negative, reflecting the negative charge of the electron beam. In contrast, the time‐resolved signals from the ACCTs were positive owing to their orientation relative to the beam's direction. To facilitate direct comparison, the BC signal was digitally inverted. The physical beam parameters of interest for each recorded pulse were the pulse width (PW), integrated charge, and peak current. To extract these parameters, the following signals were analyzed: the ACCT signal, the BC signal terminated at 13 Ω (BC_13Ω_), and the time‐derivative of the BC signal terminated at 200 kΩ (BC_200kΩ_). The 13 Ω termination resistance was chosen empirically to yield a current‐proportional signal, while the 200 kΩ (default internal oscilloscope resistance) was found to be large enough to yield a charge‐proportional signal at the relevant timescale.

Each signal was first processed through a digital 3‐MHz low‐pass filter. The PW was determined as the full width at half‐maximum (FWHM). An integration window was defined by extending the PW by 350 ns on either side of the FWHM, accounting for the ACCT signal rise time. A baseline subtraction was performed by subtracting the mean signal value from the 0.5‐µs intervals immediately before and after the integration window (Figure 1b) from the signal in the integration window. Finally, the integral of the baseline‐subtracted signal over the integration window, M, was calculated (Equations (1), (2), (3)). M is measured in V·s, also known as Wb.

(1) $$M=\int_{{\text{FWHM}}_{\text{start}}-350\;\text{ns}}^{{\text{FWHM}}_{\text{end}}+350\;\text{ns}}S\left(t\right)dt\;\left[\mu \text{Wb}\right]$$

(2) $$\begin{array}{cc} & \text{Integrated Signal ACCT}[\text{nC}]\\ & \;=M[\mu \text{Wb}]\times \text{Gain Factor}[\text{mA/V}] \end{array}$$

(3) Integrated Signal BC[μWb]=M[μWb]

A manufacturer‐supplied gain factor was applied to the ACCT signal to yield the measured charge. No gain factor was applied to the BC; therefore, the integrated signal results are reported in µWb (Equation 3). An evaluation of the BC_13Ω_ and BC_200kΩ_ signals showed that the difference in measured PW between the two acquisition modes was < 1%. The integral signal from each dataset was normalized to its own mean prior to comparison, and the residual difference was < 0.1% across the range of PWs investigated (0.5–4 µs), indicating consistent relative PW‐dependence between the charge‐accumulation and current‐viewing‐resistor acquisition modes. Therefore, given its similarity to the ACCT signals, the BC_13Ω_ signal was used for the BC signal analysis throughout this study, except for the ‘Dependency on Pulse Number’ measurement, which was done with the BC_200kΩ_ signal.

### 2.3 Irradiation conditions

The BC's response was evaluated by varying the PW, PRF, and number of pulses (N_p_) (Table 1) to test the following conditions:

1. **Dependency on Pulse Number**: 50 consecutive pulses were delivered at the highest possible charge per pulse (Q_p_) and mean beam current ($\bar{\;I)}$. (SSD = 19.3 cm, PW = 4 µs, PRF = 120 Hz, number of pulses [N_p_] = 1 – 50).
2. **Dependency on Pulse Repetition Frequency**: 3 pulses were delivered at varying PRFs. This was repeated for a range of PWs. All irradiations were repeated three times and the average across replicates recorded. (SSD = 19.3 cm, PW = 0.5–4 µs, PRF = 5–120 Hz, N_p_ = 3). The mean current $\bar{\;I\;}$ ranged from 63 to 13 000 nA.
3. **Dependency on Charge per Pulse**: Three pulses were delivered at varying PWs at 30 Hz. This was repeated at two different SSDs, resulting in IDRs of 1.4 and 0.7 MGy/s. The charge per pulse was varied by varying both the PW and the IDR, the latter of which changed the mean current in the pulse. All irradiations were repeated three times and the average across replicates recorded. (SSD = 19.3–32.3 cm, PW = 0.5–4 µs, PRF = 30 Hz, N_p_ = 3). The charge per pulse Q_p_ ranged from 4.8 to 71 nC, the mean current in the pulse $I_{p}$ ranged from 9.6 to 18 mA and the dose per pulse measured with film ranged from 0.4 to 6 Gy.

**TABLE 1:** Symbol definitions for relevant irradiation parameters used in this work.

Symbol	Description	SI Unit	Notes
N_p_	Number of pulses		
SSD	Source‐to‐surface distance	m	
PRF	Pulse repetition frequency	Hz	
PW	Pulse width	s	FWHM
Q	Charge	C	
Q_p_	Charge per pulse	C	Q / N_p_
${\bar{\;I\;}}_{p}$	Mean current in pulse	A	Q_p_ / PW
$\bar{\;I\;}$	Mean beam current	A	Q / ((N_p_ – 1)/PRF)
DPP	Dose per pulse	Gy	
IDR	Instantaneous dose rate	Gy/s	DPP/PW

For each analysis of the irradiation conditions, we included a residual plot that shows either the percent deviation of the BC signal from a linear fit to the ACCT signal, or the percent deviation of the BC/ACCT ratio from its mean value. On the residual plot, the area corresponding to a deviation of ± 3%, arbitrarily chosen for this work to denote ‘acceptably low’ deviation, is shaded to facilitate visual interpretation of the data.

## 3 RESULTS

### 3.1 Dependency on pulse number

**FIGURE 2:**
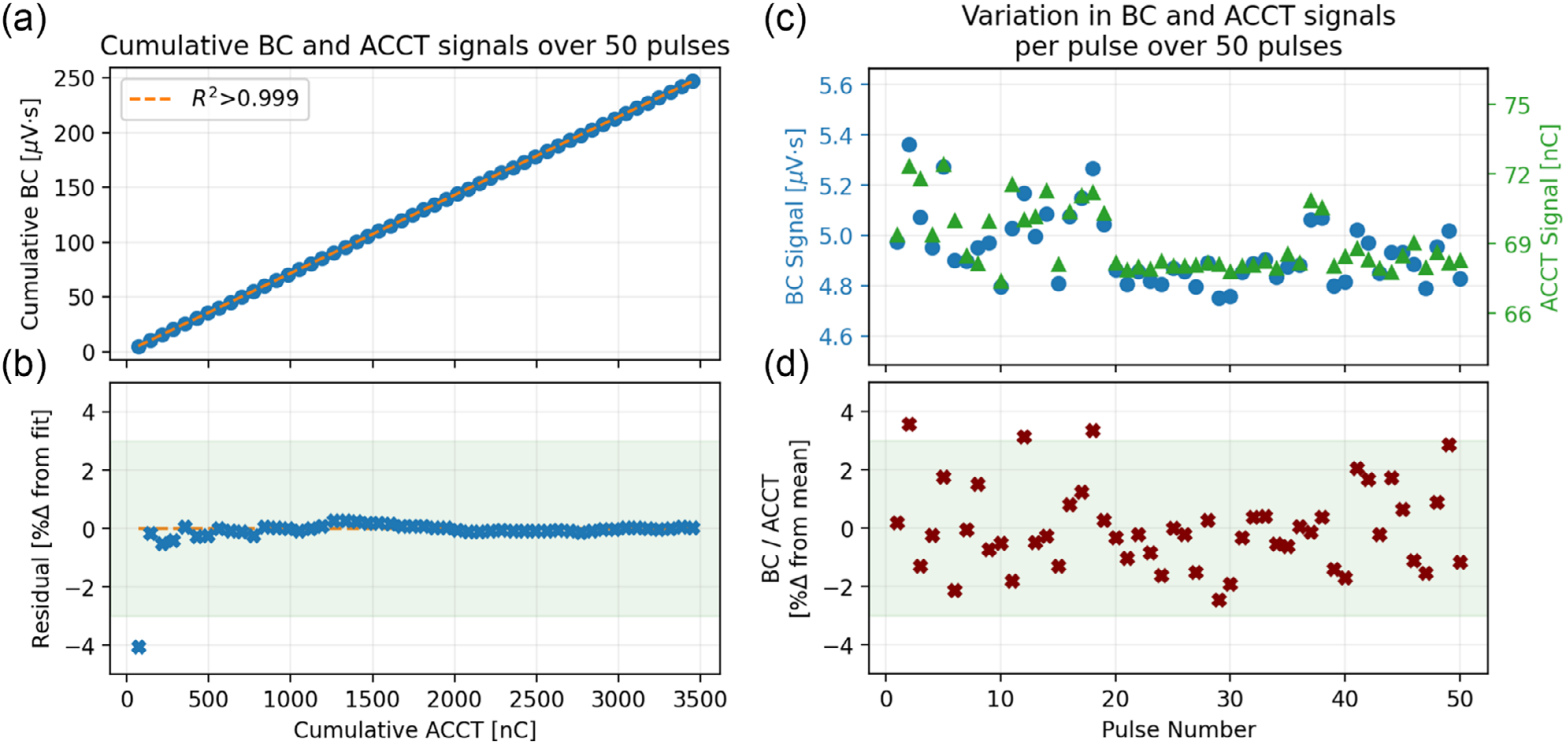
(a) Linearity of the cumulative beam collector (BC) and alternating‐current current transformer (ACCT) signals measured over 50 consecutive pulses (4 µs pulse width, 120 Hz pulse repetition frequency, 19.3 cm source‐to‐surface distance (SSD), 200 kΩ BC terminating resistance). (b) Deviation of the BC signal from a linear fit to the data. (c) BC (circles) and ACCT (triangles) signals per pulse and (d) the percent deviation of the ratio of their signals from the mean ratio value. The data shown in (c) and (d) are from the same irradiation as (a) but reflect the detectors’ signals per pulse instead of the cumulative signals over subsequent pulses. The plots in panels (a) and (b), and in panels (c) and (d), share an x‐axis.

The cumulative BC and ACCT signals were linearly proportional with a variance of less than ± 0.5% (Figure 2a,b) up to 50 pulses, excluding the first pulse. On a per‐pulse basis over the 50 pulses, the BC and ACCT signals each varied by up to ± 5%, due in part to variances in the machine output per pulse (Figure 2b). The signals of the two detectors were correlated (Pearson *R *= 0.87, *p* < 2·10^−16^) and the ratio of their signals exhibited a lower variance across the 50 pulses of less than ± 4% (Figure 2c,d).

### 3.2 Dependency on pulse width and mean dose rate

**FIGURE 3:**
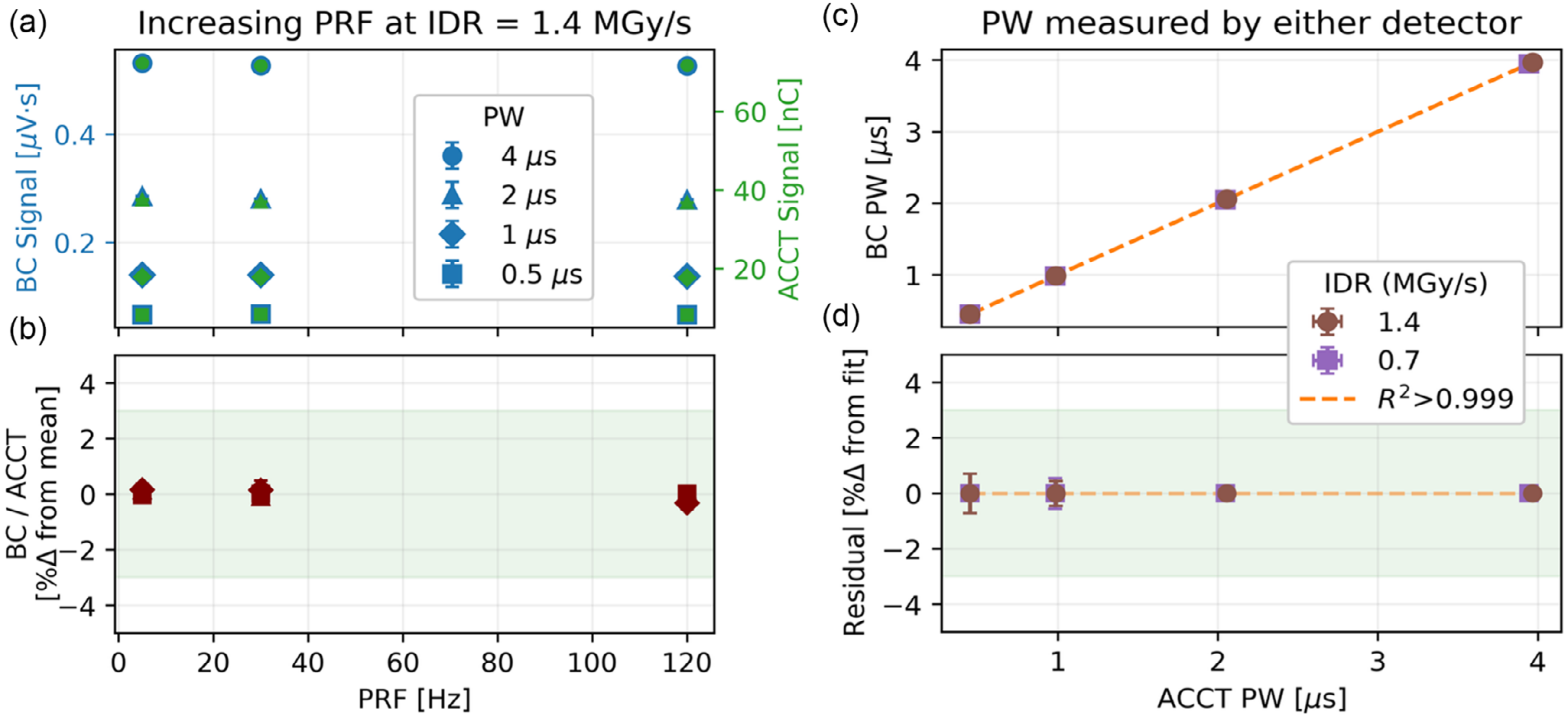
(a) Beam collector (BC) and alternating‐current current transformer (ACCT) signals as the pulse repetition frequency (PRF) is increased at various pulse widths (PWs). (b) Deviation of the BC/ACCT ratio from its mean value, evaluated for each PW independently. (c) Linearity of the pulse widths measured by the BC and ACCT signals for all nominal PW values at a source‐to‐surface distance (SSD) = 19.3 cm (Circles) and SSD = 32.3 cm (Squares), the instantaneous dose rates (IDRs) are shown for each SSD, respectively. (d) Deviation of the measured values from a linear fit. The top and bottom plots of each panel in this figure share an x‐axis.

**FIGURE 4:**
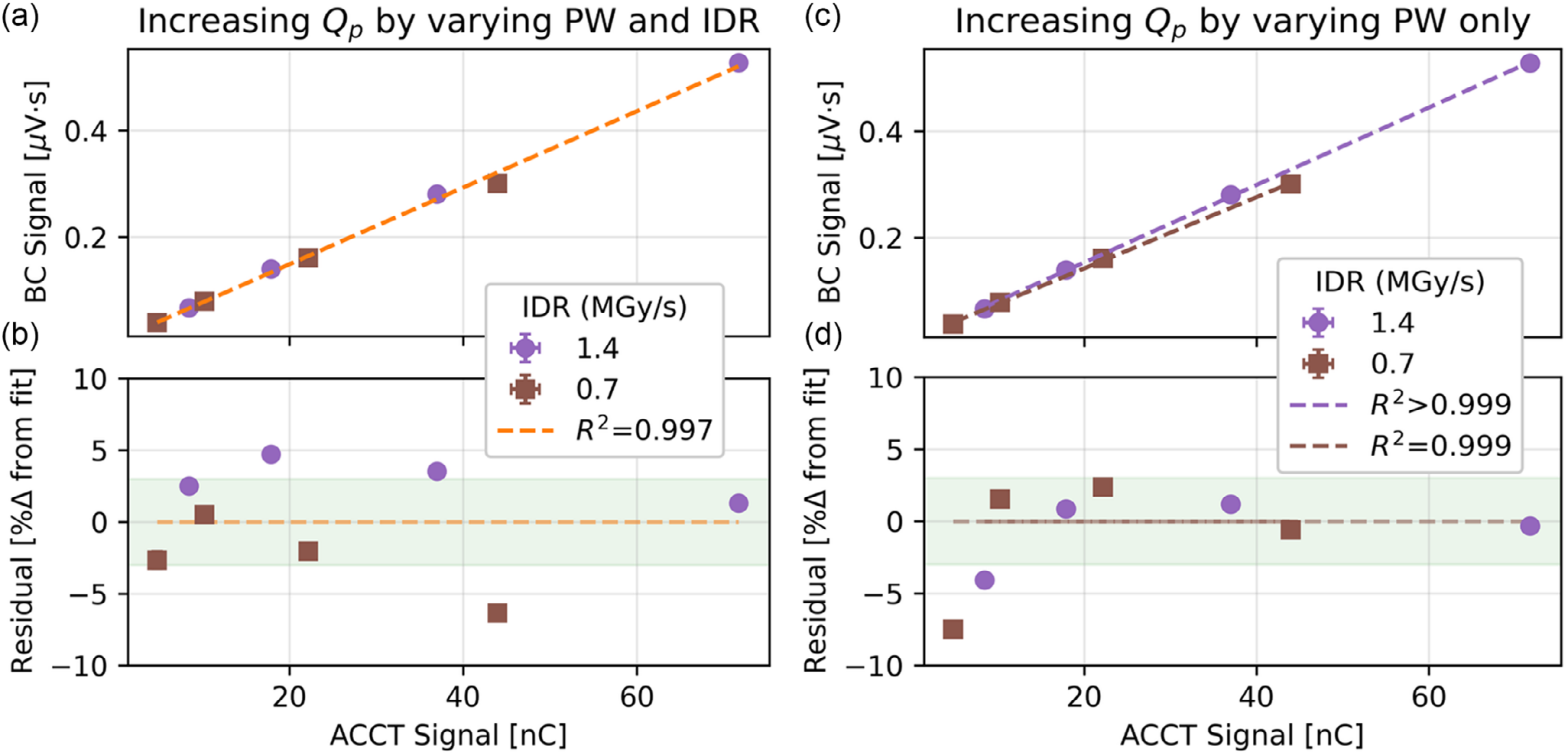
(a) Linearity of the beam collector (BC) and alternating‐current current transformer (ACCT) signals as the charge per pulse (Qp) is increased by increasing the pulse width (PW) and decreasing the source‐to‐surface distance (SSD). The signals for source‐to‐surface distance (SSD) = 19.3 cm (Circles) and SSD = 32.3 cm (Squares), but the linear fit is to the data from both SSD values together. (b) Deviation of the signal from the linear fit. The mean current in the pulse ($I_{p})$ was 9.6 mA to 18 mA, SSD = 19.3 cm and SSD = 32.3 cm, and dose‐per pulse (DPP) was 0.36 Gy to 5.8 Gy. The instantaneous dose rates (IDRs) are shown for each SSD, respectively. The top and bottom plots of each panel in this figure share an x‐axis. (c and d) Present the same data as panels a and b but with individual fit for each IDR condition.

The pulse widths measured by either detector were within ± 0.5% for all nominal PW values and for both SSD and IDR values (Figure 3). The BC signal was roughly linear with the ACCT signal as the charge per pulse was increased by increasing the PW and decreasing the IDR; the BC/ACCT ratio varied by up to ± 6% across this range (Figure 4a,b). To evaluate the BC response to only the PW (as opposed to PW and IDR together), the linearity of the BC signal with the ACCT signal for each IDR was evaluated independently (Figure 4c,d). Here, the BC/ACCT ratio varied by less than ± 3% for PW > 0.5 µs, but at PW = 0.5 µs deviated from the mean by –4% for IDR = 1.4 MGy/s and –7% for IDR = 0.7 MGy/s.

## 4 DISCUSSION

The main objective of this work was to evaluate the BC as a beam diagnostic system for UHDR electron FLASH beams. In this context, a beam diagnostic system needs to be capable of resolving the temporal structure of individual electron pulses, exhibiting a linear response with accumulated charge and charge per pulse, and be independent of the mean and instantaneous current of the beam. As has been shown elsewhere,^24^, ^25^ the ACCT has been validated for UHDR electron FLASH beams. In this work, we compared the BC against the ACCT for reference, showing that it can address the needs of a beam diagnostic system, making it a potential candidate for use as a redundant monitoring system during, for example, beam tuning, commissioning, and QA efforts. This is particularly relevant for UHDR FLASH beamlines, where accurate measurement of pulse‐level parameters such as charge per pulse and temporal beam structure is required to characterize delivery conditions associated with the FLASH effect. In this regime, several detector systems may be limited by saturation effects, insufficient temporal resolution, or constraints in beamline placement.^12^ In this context, the BC provides a complementary approach to existing systems, including ACCTs, by enabling pulse‐resolved, time‐resolved measurements of absolute charge without requiring a vacuum system, bias voltage, or external power.

From an operational perspective, the BC's performance is governed by its readout configuration. While the BC operates without a vacuum system, bias voltage, power supply, or complex electronics, its beam diagnostic performance remains fundamentally governed by the readout circuit impedance (13 Ω or 200 kΩ). This impedance influences the characteristic time constant of the device, causing it to function as a charge integrator at high impedance (via an increased time constant) and to provide time‐resolved current measurements at low impedance (via a decreased time constant). Moreover, the measured signal sensitivity increases when the BC is terminated at high impedance. For UHDR electron pulsed beams, when terminated at high impedance, the extended time constant resulted in overlapping and superimposed signals from the multiple pulses delivered. In contrast, terminating the BC at low impedance reduced measurement sensitivity but shortened the characteristic time constant, enabling the resolution of a time‐proportional current signal from individual electron pulses, like the signals observed in the ACCT. To ensure methodologic consistency and to take advantage of time‐resolved current measurements, the low impedance (13 Ω) termination was used throughout most of this work (Figure 1b). The high impedance (200 kΩ) configuration was used specifically for the Dependency on Pulse Number measurement. Because each of the 50 pulses was captured and analyzed as a separately triggered acquisition rather than as a single continuous multi‐pulse waveform, the inter‐pulse overlap described above did not apply, allowing the per‐pulse signal to be obtained directly from a single voltage difference rather than by numerically integrating a time‐resolved current trace. This substitution had no effect on the reported result, as the two configurations were shown to be equivalent in PW (< 1%) and mean‐normalized integral signal (< 0.1%) across the PW range investigated.

The results from the irradiations demonstrated the following.

- The BC maintains a linear response under realistic beam‐monitoring conditions for UHDR electron FLASH multi‐pulse deliveries. The BC response was linear with respect to the number of electron pulses delivered and total accumulated charge, even at high output settings where 50 consecutive pulses were delivered at the highest charge per pulse (72 nC/pulse) and mean current (13 000 nA). Using the ACCT signal as a reference for charge measurements, the BC demonstrated excellent charge linearity (Figure 2a). The deviation from linearity improves with increasing charge as the per‐pulse variations in the detectors’ relative signals average out, which explains the higher variance in the first pulse (Figure 2b). These per‐pulse variations are showcased in Figure 2d, and are likely a combination of inherent uncertainties in either detector, as well as slight per‐pulse variations in beam parameters, like energy, inherent to the linac. Per‐pulse variations in the beam output, visible in Figure 2c, are accounted for by always comparing the BC signal relative to the ACCT signal.
- The BC response was unaffected by changes in the PRF of the beam. The PRF was adjusted from 5 to 120 Hz and PWs from 0.5 µs to 4 µs, resulting in BC mean currents ranging from 63 to 13 000 nA. As shown in Figure 3a,b, the BC response showed no dependency on the PRF, which remained consistent across the tested range and was affected only by the PW. Moreover, the BC pulse width measurements agreed with those of the ACCT (Figure 3c,d), showing its temporal resolution capabilities.
- The BC showed an approximate linear response with the amount of charge in each electron pulse as recorded by the ACCT. The PW was adjusted from 0.5 µs to 4 µs (nominal), and two IDRs were evaluated (IDR = 1.4 MGy/s cm and IDR = 0.7 MGy/s), resulting in Qp ranging from 0.3 to 71 nC, $I_{p}$ of 1.4 to 18 mA, and DPP of 0.36 Gy to 5.6 Gy. Figure 4a shows that when Qp is changed by varying both pulse width and IDR, a linear correlation was found when performing a combined linear fit of the measured Qp for both IDRs with their respective PWs (Figure 4a). Overall variability was 5% for IDR = 1.4 MGy/s and 7% for IDR = 0.7 MGy/s.
  From a qualitative perspective, the residuals exhibit a relative increase for ACCT signals below 20 nC, followed by a relative decrease for ACCT signals above 20 nC. This trend is consistent for both IDRs, despite differences in magnitude (Figure 4b **)**. Although the underlying cause of this trend remains unclear, a potential explanation for the observed variability—also considered a limitation of this study—is the presence of slight deviations in the physical setup. This can come from the fact the BC received more radiation than the ACCT because of scattered radiation impinging on its exposed surfaces outside the collimation ring (Figure S1).

Another limitation of this study lies in the area of the BC exposed to radiation. Measurements were limited to the beam transmitted through a 4.5‐cm diameter collimator and aligned with the ACCT, enabling a direct comparison between both devices. No other field sizes were evaluated. As a result, the BC's response as a function of field size remains uncharacterized, and additional studies are required to assess its performance under varying field sizes.

Finally, potential non‐linear behavior associated with accumulating charge in the BC was not observed. The system was tested with up to 50 consecutive pulses at the highest achievable Q_p_ and $I_{p}$, which we consider to represent a robust test scenario within the expected clinical and preclinical application range. Nevertheless, further investigations involving higher pulse counts or greater accumulated charge will be required to characterize the detector's non‐linear response threshold.

## 5 CONCLUSIONS

We evaluated the beam diagnostic performance of a Faraday cup beam collector (BC) on a UHDR electron beamline across a range of irradiation parameters relevant to FLASH‐RT. We compared the performance of the BC with that of an ACCT, previously shown to be linear with beam output and independent of other irradiation parameters.^25^ Like the ACCT, the BC provided real‐time, highly time‐resolved signals of the beam current, with no apparent differences in the measured pulse shapes. Relative to the ACCT, the BC signal per pulse varied by less than ± 2% over a delivery of 50 pulses at the highest beam output parameters available. No PRF dependency was observed in the tested range from 5 to 120 Hz. At PW ≥ 1 µs, the BC and ACCT signals deviated by less than 3%. The BC achieves highly time‐resolved measurements of pulsed UHDR electron beams and related UHDR beam parameters, which makes it a potential candidate diagnostics system for preclinical and clinical scenarios for UHDR electron FLASH.

## AUTHOR CONTRIBUTIONS

*Conceptualization and design*: Alan Lopez, Alexander Baikalov, Kevin Liu, Nolan Esplen, Stefan Bartzsch, and Emil Schüler. Data collection: Alexander Baikalov, Alan Lopez, and Kevin Liu. *Data analysis*: Alexander Baikalov and Alan Lopez. *Manuscript drafting*: Alan Lopez and Alexander Baikalov. *Edits and final approval of manuscript*: Alan Lopez, Alexander Baikalov, Kevin Liu, Nolan Esplen, Stefan Bartzsch, and Emil Schüler.

## FUNDING INFORMATION

Research reported in this publication was supported in part by the National Cancer Institute of the National Institutes of Health under Award Number R01CA266673; by the University Cancer Foundation via the Institutional Research Grant program at MD Anderson Cancer Center; by a grant from MD Anderson's Division of Radiation Oncology; by Cancer Center Support (Core) Grant P30 CA016672 from the National Cancer Institute of the National Institutes of Health, to The University of Texas MD Anderson Cancer Center [PI : PW Pisters]; and by the Andrew Sabin Family Foundation Fellowship at the University of Texas MD Anderson Cancer Center. The content is solely the responsibility of the authors and does not necessarily represent the official views of the National Institutes of Health.

## CONFLICT OF INTEREST STATEMENT

The authors declare no conflicts of interest.

## Supporting information




**Supporting Information**: acm270819‐sup‐0001‐FigureS1.docx

## Data Availability

The raw data supporting the conclusions of this article will be made available by the authors upon reasonable request.
