# Supplementary material for: Evaluation of a Faraday cup‐style detector as a beam diagnostic system for ultra‐high dose rate (FLASH) electron beams
Source: J Appl Clin Med Phys. 2026 Sep 24;27(10):e70819. doi: 10.1002/acm2.70819 (PMC13612965; doi:10.1002/acm2.70819)
Supplement: Supplementary file 1 [file ACM2-27-e70819-s001.docx]

**Supplementary Materials**

***Figure S1.*** *Experimental setup of the beam collector (BC) and alternating-current current transformer (ACCT) under the linac treatment head at an extended source-to-surface distance (SSD) (pictured here, SSD = 32.3 cm). The beam arrives from the top, passing through a stack of collimators of decreasing diameter ending with the 4.5-cm collimator, which is directly atop the ACCT in all measurements. After the collimators, the beam passes through the 5.5-cm* ACCT *and finally impinges on the front surface of the BC. The ACCT is separated from the surface of the BC by two 2-mm-thick plastic blocks (light blue).*
